# Better Understanding in the Differentiation of Thyroid Follicular Adenoma, Follicular Carcinoma, and Follicular Variant of Papillary Carcinoma: A Retrospective Study

**DOI:** 10.1155/2014/321595

**Published:** 2014-09-18

**Authors:** Jung Hyun Yoon, Eun-Kyung Kim, Ji Hyun Youk, Hee Jung Moon, Jin Young Kwak

**Affiliations:** Department of Radiology, Severance Hospital, Research Institute of Radiological Science, Yonsei University College of Medicine, 50 Yonsei-ro, Seodaemun-gu, Seoul 120-752, Republic of Korea

## Abstract

*Background.* To evaluate the role of ultrasonography (US), US-guided fine-needle aspiration (USFNA) and intraoperative frozen section (FS) in follicular neoplasm. *Methods*. US features, USFNA cytology, and FS results were compared based on the pathology results of patients with follicular adenoma (FA), follicular carcinoma (FC), and follicular variant of papillary thyroid carcinoma (FVPTC). *Results*. FC and FVPTC showed significantly higher rates of suspicious US features (*P* < 0.05) and positive findings on either US or cytology, 80.0% and 90.7%, compared to FA, 64.5% (*P* = 0.001). Intraoperative FS showed higher malignant rates in FVPTC and FC (81.8% and 75.0%, resp.), compared to FA (3.8%, *P* < 0.001). *Conclusion*. Suspicious US features were more significantly seen in FC and FVPTC compared to FA. Intraoperative FS is useful in the differential diagnosis of these lesions and supplements cytology results of USFNA.

## 1. Introduction 

Thyroid nodules showing follicular morphologic features include adenomatous nodule, follicular adenoma (FA), follicular carcinoma (FC), and follicular variant of papillary thyroid carcinoma (FVPTC) [1]. Cytologic features are known to overlap among these tumors [2, 3], and definite diagnosis of FA, FC, and FVPTC is mostly obtained by pathologic examination following complete excision of the lesion [1, 4, 5].

The diagnosis of a solitary, encapsulated nodule with follicular histology features is frequently problematic since a broad range of benign to malignant subtypes of follicular tumors need to be differentiated, such as FA, FC, and FVPTC [6]. Differential diagnosis of FC from FA is based on the presence of capsular, vascular, or extrathyroidal tissue invasion, and nodal or distant metastasis [4, 6, 7]. Diagnosis of follicular neoplasm based on cytology alone has always been challenging to both clinicians and cytopathologists, since it is well known that cytologic features overlap in both benign follicular adenoma and carcinomas [2, 3, 8, 9]. Studies have investigated ways in providing additional information that may be helpful in differential diagnosis and surgical planning of follicular neoplasm [4, 6, 10, 11] but controversy still remains and clinicians are still skeptical until they see the conclusive reports on permanent section.

Diagnostic criteria for the cytologic diagnosis of FVPTC are in general similar to those of PTC, that is, cells containing fine chromatic, nuclear grooves, intranuclear inclusions, and overlapping nuclei [12–15], but FVPTC lacks papillary groups and shows follicular patterns with variable colloid component, which can also be seen in benign and neoplastic follicular lesions [1, 13]. This overlap makes accurate cytologic diagnosis difficult in FVPTC and results in the low sensitivity (25% to 42%) of fine-needle aspiration (FNA) in the diagnosis of FVPTC, compared to conventional papillary carcinoma (sensitivity range from 60% to over 90%) [13–15].

False-negative cytologic results are also occasionally observed, for example, follicular carcinomas containing macrofollicular pattern with abundant background colloid can be easily mistaken as a benign adenomatoid colloid nodule on cytology [16]. Even with surgery, differential diagnosis between FA, minimally invasive FC, and FVPTC in a solitary, encapsulated nodule showing follicular histology has been problematic [6]. While there are several studies focusing on ways to differentiate these neoplasms [5, 7, 15, 16], little has been evaluated in association between the ultrasound (US) features or the cytology results of USFNA within these tumors. In this study, we evaluated the differences in US features and the role of US-guided fine-needle aspiration (USFNA) and intraoperative frozen section (FS) in FA, FC, and FVPTC.

## 2. Materials and Methods

This retrospective study was approved by the institutional review board (IRB) of Severance Hospital, Yonsei University, Seoul, Republic of Korea. Neither patient approval nor informed consent was required for review of medical records or images. Informed consent was signed and obtained from all patients before USFNA or surgery prior to procedures.

### 2.1. Study Population

From January 2003 to December 2008, our institutional database was reviewed for patients diagnosed with FA, FC, and FVPTC after surgical excision. A total of 281 patients with 282 thyroid nodules were included in this study. Among them, 51 patients were excluded because they had either undergone USFNA at an outside clinic or had not undergone preoperative cytologic diagnosis. In total, 230 patients with 231 thyroid nodules were included in this study. Of the 230 patients, 45 (19.6%) were men, and 185 (80.4%) were women. Mean age of the 230 patients included was 44.0 years. Mean size of the 231 thyroid nodules was 27.3 mm. Medical records, US images and radiological reports, and cytopathologic reports of these patients were reviewed, retrospectively.

### 2.2. US Imaging and Imaging Analyses

US was performed in all patients using a 7–15 MHz linear array transducer (HDI 3000 or 5000; Philips Medical Systems, Bothell, WA) or a 5–12 MHz linear array transducer (iU22; Philips Medical System). Compound imaging was obtained in all images using HDI5000 or iU22 machines.

Real-time US was performed by 1 of the 5 board-certified radiologists with 1–13 years of experience in thyroid imaging. US features of the thyroid nodules were retrospectively reviewed and analyzed by one dedicated thyroid radiologist (Y.J.H) with 3 years of experience. The radiologist was blinded to the clinical and cytopathological information of the patient during image review. US features of each thyroid nodule were described according to internal components, echogenicity, margin, calcifications, and shape [5]. Internal components were divided into solid nodules, mixed solid, and cystic nodules, that is, mainly solid nodules containing more than 50% of solid contents, mainly cystic nodules containing less than 50% of solid contents, and cysts. Echogenicity was divided into hyper or isoechoic (nodules showing hyperechogenicity or isoechogenicity compared with the adjacent normal thyroid parenchyma), hypoechoic (nodules showing hypoechogenicity compared to the adjacent normal thyroid parenchyma), and markedly hypoechoic (nodules showing hypoechogenicity compared to the adjacent strap muscle). Margin was classified as circumscribed or noncircumscribed (i.e., microlobulated or irregular margins). Calcifications were classified as microcalcifications (tiny, punctate, echogenic foci measuring less than 1 mm) [17] or mixed microcalcifications with macrocalcifications, macrocalcifications (including eggshell calcifications), and no calcifications. Shape was divided into parallel or nonparallel (greater in the anteroposterior dimension than the transverse dimension, or “taller-than-wide”). Malignant US features were defined as marked hypoechogenicity, noncircumscribed margins, microcalcifications or mixed calcifications, and nonparallel shape, based on previously published criteria [18]. Final assessments of the thyroid nodules were given as probably benign (when none of the suspicious US features described above was present) or suspicious malignant (when 1 or more suspicious US features above were present).

### 2.3. USFNA and Cytological Analyses

USFNA was subsequently performed by the same radiologist who obtained the real-time US images. USFNA was performed either on the thyroid nodules showing suspicious US features or on the nodule with the largest size without any suspicious US features.

USFNA was performed at least twice from the targeted thyroid nodule using a 23-gauge needle attached to a 20 mL disposable syringe with an aspirator or a 23-gauge needle attached to a 2 mL disposable syringe without an aspirator, depending on the radiologist's preference. Local anesthesia was not routinely applied. Aspirated material was expelled on to glass slides, smeared, and immediately placed in 95% alcohol for Papanicolaou staining. The remaining material in the syringe was rinsed in normal saline for cell block processing. The cytopathologists were not present during USFNA procedures, and additional staining was performed on a case-by-case basis at the request of the cytopathologist.

One of the 5 cytopathologists specializing in thyroid pathology interpreted the slides obtained from USFNA. During the study period, cytologic reports were divided into the following categories: (1) malignant, (2) suspicious for malignant, (3) indeterminate, (4) benign, and (5) inadequate [5, 19–21]. Malignancy indicated specimen showing abundant cells with unequivocal cytologic features of malignancy. Suspicious for malignancy was used in specimen showing cytologic atypia, that is, crowded, overlapping, pleomorphic, and enlarged nuclei, but with insufficient cellularity for definite diagnosis of malignancy [19, 21]. Indeterminate cytology included follicular neoplasm and Hürthle cell neoplasm, indicating specimen showing monotonous cellular population, scanty colloid, and lacking papillary carcinoma features [22]. Benign cytology includes colloid nodules, nodular hyperplasia, lymphocytic thyroiditis, Graves' disease, and postpartum thyroiditis. Inadequate cytology indicates specimen showing less than the required minimum of six groupings of well-preserved thyroid cells, each consisting of less than 10 cells per group [19, 20].

### 2.4. Surgical Procedures and Intraoperative Frozen Section

The extent of surgery was performed based on the cytology results and US features. A lobectomy, subtotal thyroidectomy, or total thyroidectomy was performed if cytology findings were diagnosed as malignancy or suspicious for malignancy or if the US features were assessed as suspicious malignant in nodules with benign cytology diagnosis. A lobectomy, or subtotal thyroidectomy, was performed if the cytology results were benign. Of the cytology results was inadequate or indeterminate, the extent of thyroid surgery was based on intraoperative FS during surgery.

Tissue samples including the thyroid nodule and/or the adjacent thyroid parenchyma were obtained and processed for FS analyses. Frozen tissue samples were subsequently cut and stained for diagnosis. After diagnosis was made, results were notified to the operation room. Diagnosis was classified into the following 3 categories in FS: (1) malignant, (2) benign, and (3) deferred results, including follicular neoplasm [5, 21].

### 2.5. Statistical Analyses

Histopathologic results from surgery were considered standard reference. In comparison to the mean age and mean size of thyroid nodules on US among the three neoplasms, Analysis of variance (ANOVA) test and post hoc test was used. *χ* ^2^-test or Fisher's exact test was used in comparison to US features among the final pathology of the disease. Diagnostic performances including sensitivity, specificity, positive predictive value (PPV), negative predictive value (NPV), and accuracy were calculated for USFNA cytology and intraoperative FS results. In regard to USFNA, inadequate cytology was excluded during calculation of diagnostic performances, considering benign cytology as negative and indeterminate, suspicious for malignancy, and malignant cytology as positive. For comparison with intraoperative FS, diagnostic performances of USFNA excluding both inadequate and indeterminate cytology were also calculated. In regard to FS, deferred results were excluded when obtaining diagnostic performances [5].

*P* value of less than 0.05 was considered significant. Statistical analysis was performed by the SAS system (MAGREE SAS Macro program; SAS Institute, Cary, NC).

## 3. Results

Of the 231 thyroid lesions, 152 (65.8%) were diagnosed as FA, 25 (10.8%) as FVPTC, and 54 (23.4%) as FC on surgical pathology. Mean age and size among the three neoplasms are summarized in Table 1. Mean age of the nodules diagnosed as FC was the oldest, 47.2 ± 17.7 years, with statistical significance (*P* = 0.042). When comparing FC to FA, mean age was also significantly older in FC (*P* = 0.034). No significant differences were observed in mean age when comparing between FVPTC and FA or between FVPTC and FC (*P* = 0.101 and 0.991, resp.). Mean size of the nodules diagnosed as FVPTC was the smallest, 16.3 ± 14.6 mm, with statistical significance (*P* < 0.001). FVPTC was significantly smaller than FA, 29.7 ± 14.5 mm to 16.3 ± 14.6 mm (*P* < 0.001), but tumor size between FC and FA did not show statistical significance (*P* = 0.126).

US features of the 231 thyroid nodules are summarized in Table 2. Of the 152 nodules diagnosed as FA, 136 (89.5%) showed no suspicious US features. In contrast, 12 (48.0%) of the 25 nodules diagnosed as FC and 28 (51.9%) of the 54 nodules diagnosed as FVPTC showed one or more suspicious US features. Lesions diagnosed as FC and FVPTC showed significantly higher rates of suspicious US features compared to FA (*P* < 0.001). Suspicious US features such as hypoechogenicity or marked hypoechogenicity, noncircumscribed margins, presence of micro- or macrocalcifications, or nonparallel orientation were significantly associated with FC or FVPTC than FA (*P* < 0.05).

**Figure 1:**
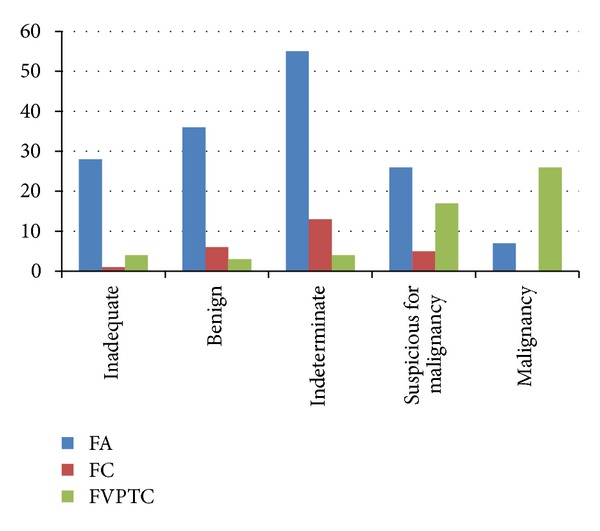
Results of USFNA cytology of the 231 thyroid nodules. Note: numbers in image represent percentages (%).

Results of USFNA cytology are summarized in Table 3 and Figure 1. Rate of inadequate cytology on USFNA was higher in FA (18.4%) compared to FC (4.0%) and FVPTC (7.4%). Also, rate of benign and indeterminate cytology was relatively higher in FA (23.7% and 36.2%) and FC (24.0% and 52.0%) compared to FVPTC (5.6% and 7.4%, resp.). In contrast, rate of suspicious for malignancy and malignant cytology was higher in FVPTC (31.5% and 48.1%) than FA (17.1% and 4.6%) or FC (20.0% and 0.0%, resp.). When considering each type of neoplasm, 88 of 152 (57.9%) nodules diagnosed as FA, 18 of 25 (72.0%) nodules diagnosed as FC, and 47 of 54 (87.0%) nodules diagnosed as FVPTC were diagnosed as indeterminate, suspicious for malignancy or malignancy on cytology, showing statistical significance (*P* < 0.001).

**Figure 2:**
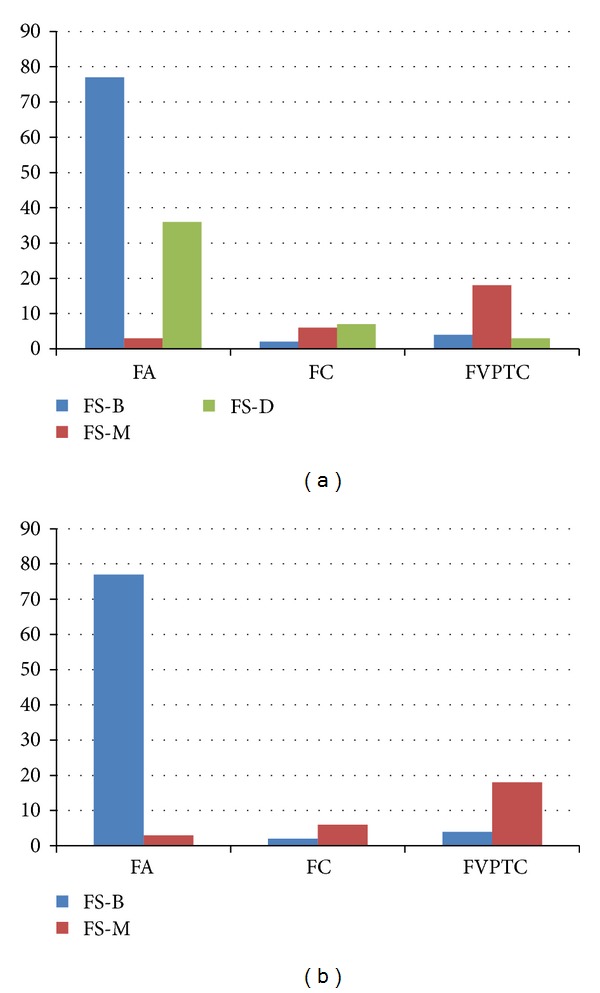
Results of intraoperative frozen section of the 231 thyroid nodules. Note: numbers in image represent percentages (%).

Of the 231 thyroid nodules, 156 (67.5%) underwent intraoperative FS (Table 3, Figure 2). Among them, 46 (29.5%) were deferred to final pathology. Malignant results on intraoperative FS significantly correlated to FC or FVPTC on final pathology (*P* < 0.001). Two of the 15 nodules diagnosed as FC and 4 of the 25 nodules diagnosed as FVPTC showed false-negative results on intraoperative FS. Five of the 6 (83.3%) nodules showing false-negative FS were diagnosed as suspicious for malignancy or malignancy on USFNA. Also, 3 of the 116 nodules diagnosed as FA showed false-positive results on intraoperative FS.

Diagnostic performances of USFNA and intraoperative FS are summarized in Table 4. Specificity of USFNA was low, 29.3%, when considering indeterminate cytology as positive. Overall diagnostic performances of intraoperative FS were higher than USFNA.

## 4. Discussion

Follicular adenomas are well-encapsulated thyroid neoplasms which do not show the typical invasiveness of follicular carcinoma, nor abnormal nuclear features of papillary carcinomas [7]. FA and FC, along with FVPTC, are well-encapsulated lesions, sharing many imaging and cytologic features, and show relatively benign US features [7, 23, 24]. In our study, tumor size of FVPTC was significantly smaller than FC or FA, 16.3 mm to 36.4 mm and 29.7 mm, respectively. As mentioned above, thyroid lesions of follicular pattern tend to represent more benign features on US and, therefore, may have not undergone diagnostic procedures such as USFNA unless they have reached sizes over 10 mm or until they have grown to sizes that may have brought about clinical significance such as presence of symptoms.

Common suspicious US features such as microlobulated or irregular margins, marked hypoechogenicity, taller-than-wide shape, and presence of microcalcifications are used in differentiating papillary thyroid carcinoma with high diagnostic accuracy but do not seem to work the same when differentiating between lesions of follicular patterns [10]. US features reported for follicular neoplasm or FVPTC are relatively benign appearing, showing well-defined, solid mass with oval shape, surrounding hypoechoic rim [10, 15, 23, 25], among which findings do not significantly differ between benign FA or malignant FC or FVPTC. Our results showed that 52.0% of FC and 48.1% of FVPTC had no suspicious US features, consistent with other reports in that malignant form of follicular neoplasm has relatively benign appearance on US. However, several suspicious US features of papillary thyroid carcinoma such as microlobulated or ill-defined margins, microcalcifications, and taller-than-wide shape have been reported to be more significantly seen in the malignancy among nodules showing indeterminate cytology [5]. In our results, suspicious US features were significantly associated with FC or FVPTC than FA. Although FC or FVPTC do not show the typical suspicious US features as frequently as conventional PTC, the presence of each individual US features may have a role in leading the radiologists or clinicians in differentiating these lesions from FA.

While USFNA is widely used in discriminating between benign and malignancy in various lesions of the thyroid showing excellent performances (sensitivity 65%–98%, specificity 72%–100%) [3, 5, 26, 27] this has limited value in the differential diagnosis of follicular neoplasm, in which USFNA is considered only as a “screening test” [28]. Nodules diagnosed as follicular neoplasm or suspicious for follicular neoplasm on cytology mostly undergo surgery for diagnostic purposes, but the true role of USFNA cytology results in predicting diagnosis of follicular neoplasm has not been clarified. Indeed, sensitivity of USFNA in the diagnosis of FVPTC has been reported to be lower than PTC, ranging from 25.0% to 32.0% [13, 15, 29, 30]. Cytologic diagnosis of follicular patterned lesions of the thyroid with USFNA is imprecise, although one can predict a diagnosis but cannot reach a final conclusion based on cytology alone [1]. Results of our study showed higher rates of benign cytology in FA (23.7%) and FC (24.0%) compared to FVPTC (5.6%). Cytology specimen showing multinodular process with intervening colloid-rich thyroid tissue is often seen not only in follicular neoplasm but also in benign adenomatoid nodules [1, 31], which may have been a cause for false-negative results. Another cause for benign cytology results in FC may be failure to sample in FC with cystic areas [32]. Nearly 32.0% of FC included in our study revealed cystic portions within the tumor on preoperative US, which may have been one of the causes for benign results on USFNA.

The diagnosis rate of FVPTC on USFNA cytology is low in clinical practice, ranging from 9.0% to 36.0% [13, 33, 34]. Unlike conventional papillary carcinoma, the presence of abundant colloid, subtle nuclear features of papillary carcinoma, and the absence of papillary formations and psammomatous bodies are the known causes that interfere with the definite diagnosis on cytology [22, 32]. But a recent study suggested that some cytologic features of conventional PTC such as fine chromatic, nuclear grooving, and intranuclear inclusions are present at high frequency in FVPTC [13]. Although present with a wide variance, these specific features may help in classifying FVPTC towards indeterminate or suspicious for papillary carcinoma which is enough to lead towards surgical management [13]. Our study showed higher rates of suspicious for malignancy or malignant cytology results in FVPTC (31.5% and 48.1%) than FA (17.1% and 4.6%) or FC (20.0% and 0.0%), and the cytology features of FVPTC mentioned above may have contributed to these results. In addition, cystic changes, hemorrhage, and degeneration of collagen can be found in FA [35–37], and along with the typical “spoke and wheel” vascularity pattern characteristic for FA may have been the causes for high rates of inadequate cytology (18.4%) compared to FC (4.0%) and FVPTC (7.4%) [37, 38].

Intraoperative FS has been popularly used in the diagnosis of thyroid nodules, having an important role in deciding the surgical extent based on its results [39, 40]. Although it is not useful in the differential diagnosis of benign to malignant thyroid nodules [21, 41], it is often used as a supplement to preoperative USFNA. Controversy remains in the role of intraoperative FS in follicular neoplasm. Some proved increased specificity, but lower sensitivity compared to USFNA, diagnostic accuracy ranging from 50% to 98% [5, 42–44], while others claim that FS does not effectively provide any additional information in the diagnosis of follicular neoplasm [45]. In one study on USFNA and FS, both FNA and FS were highly accurate in predicting final pathology when the diagnosis was papillary carcinoma or benign but missed 44% of the malignancies in follicular lesions [39]. Diagnostic performances of intraoperative FS when excluding the deferred results in our study showed high sensitivity (80.0%), specificity (96.3%), and accuracy (91.8%), showing better performances than USFNA as in a recent report [44]. FVPTC and FC showed significantly higher malignant results in intraoperative FS, 81.8% and 75.0%, respectively, compared to FA, 3.8%. These results are similar to a previous study suggesting that with intraoperative FS, nearly 52% to 60% of the malignant subtype of follicular neoplasm do not require secondary procedures [44]. Also, among the nodules showing false-negative intraoperative FS results, 83.3% (5 of 6 diagnosed as benign on FS, Table 3) were diagnosed as suspicious for malignancy or malignancy on USFNA, which further supports the complementary relation of USFNA and intraoperative FS in lesions of follicular pattern in thyroid [39].

There are several limitations to our study. First, this study was in a retrospective design, including patients diagnosed as FA, FC, or FVPTC on surgery. Selection bias may have existed in patient inclusion. Second, 5 cytopathologists were involved in interpretation of cytology, intraoperative FS, and final pathologic diagnosis. Observer variability on the diagnosis of follicular neoplasm may have affected the results of our study [1]. Third, vascularity of the nodule on Doppler US was not considered in the analysis of US features among the subtypes of follicular neoplasm included in this study. Controversy remains in the role of vascularity on US in the diagnosis of thyroid nodules [46, 47], and how it would apply to follicular neoplasm is yet to be explained.

In conclusion, suspicious US features were more significantly seen in FC and FVPTC compared to FA. Intraoperative FS is useful in the differential diagnosis of these lesions and supplements cytology results of USFNA.

## Figures and Tables

**Table 1:** Comparison of mean age and size among the 231 thyroid nodules diagnosed as follicular adenoma, follicular carcinoma, and follicular variant of papillary thyroid carcinoma.

Pathology	N	Age (years)			P	Size (mm)			P
		Mean ± SD	Min	Max	0.042	Mean ± SD	Min	Max	<0.001
FA	152	42.5 ± 12.8	19.0	72.0	—	29.7 ± 14.5	6.0	73.0	—
FC	25	47.2 ± 17.7	15.0	78.0	0.230∗	36.4 ± 20.2	13.0	100.0	0.126∗
FVPTC	54	46.9 ± 9.9	27.0	64.0	0.101∗	16.3 ± 14.6	3.0	100.0	<0.001∗

FA: follicular adenoma.

FC: follicular carcinoma.

FVPTC: follicular variant papillary thyroid carcinoma.

*N*: number of cases.

SD: standard deviation.

∗values when compared to follicular adenoma.

**Table 2:** Comparison of US features among the 231 thyroid nodules diagnosed as follicular adenoma, follicular carcinoma, and follicular variant of papillary thyroid carcinoma.

US features	Pathology			Total	P
	FA (n = 152)	FC (n = 25)	FVPTC (n = 54)		
Composition					0.033
Solid	110 (72.4)	17 (68.0)	49 (90.7)	176 (76.2)	
Mainly solid	38 (25.0)	6 (24.0)	4 (7.4)	48 (20.8)	
Mainly cystic	4 (2.6)	2 (8.0)	1 (1.9)	7 (3.0)	
Echogenicity					<0.001
Hyper/isoechoic	94 (61.8)	9 (36.0)	12 (22.2)	115 (49.8)	
Hypoechoic	58 (38.2)	14 (56.0)	42 (77.8)	114 (49.3)	
Markedly hypoechoic	0 (0.0)	2 (8.0)	0 (0.0)	2 (0.9)	
Margin					<0.001
Circumscribed	143 (94.1)	20 (80.0)	30 (55.6)	193 (83.5)	
Noncircumscribed	9 (5.9)	5 (20.0)	24 (44.4)	38 (16.5)	
Calcifications					<0.001
Micro- or mixed	1 (0.7)	3 (12.0)	7 (13.0)	11 (4.8)	
Macro- or eggshell	12 (7.9)	4 (16.0)	13 (24.0)	29 (12.6)	
Negative	139 (91.4)	18 (72.0)	34 (63.0)	191 (82.6)	
Shape					0.006
Parallel	149 (98.0)	23 (92.0)	47 (87.0)	219 (94.8)	
Nonparallel	3 (2.0)	2 (8.0)	7 (13.0)	12 (5.2)	
Final assessment					<0.001
Probably benign	136 (89.5)	13 (52.0)	26 (48.1)	175 (75.8)	
Suspicious malignant	16 (10.5)	12 (48.0)	28 (51.9)	56 (24.2)	

Note: percentages are in parentheses.

**Table 3:** Correlation of USFNA cytology and intraoperative frozen section results to final pathology.

Cytology	*N* (%)	Pathology											
			FA				FC				FVPTC		
		Total	FS-B	FS-D	FS-M	Total	FS-B	FS-D	FS-M	Total	FS-B	FS-D	FS-M
			*n* = 116∗				*n* = 15^†^			* n* = 25^‡^		
Inadequate	33 (14.3)	28 (18.4)	18	6	0	1 (4.0)	1	0	0	4 (7.4)	0	0	3
Benign	45 (19.5)	36 (23.7)	16	2	1	6 (24.0)	0	3	0	3 (5.6)	0	0	0
Indeterminate	72 (31.2)	55 (36.2)	25	22	0	13 (52.0)	0	4	5	4 (7.4)	0	0	3
Suspicious for malignancy	48 (20.8)	26 (17.1)	14	6	2	5 (20.0)	1	0	1	17 (31.5)	2	3	6
Malignancy	33 (14.2)	7 (4.6)	4	0	0	0 (0.0)	0	0	0	26 (48.1)	2	6	0

Total	231	152	77 (66.4)	36 (31.0)	3 (2.6)	25	2 (13.3)	7 (46.7)	6 (40.0)	54	4 (16.0)	3 (12.0)	18 (72.0)
Total excluding defer	110	80	77 (96.3)	—	3 (3.8)	8	2 (25.0)	—	6 (75.0)	22	4 (18.2)	—	18 (81.8)

Percentages are in parentheses.

FS-B: benign on frozen section.

FS-D: deferred on frozen section.

FS-M: malignancy on frozen section.

∗36 cases without FS excluded.

^†^10 cases without FS excluded.

^‡^29 cases without FS excluded.

**Table 4:** Diagnostic performances of USFNA and intraoperative frozen section.

(%)	FNA∗	FNA^†^	FS^‡^
Sensitivity	87.8 (65/74)	84.2 (48/57)	80.0 (24/30)
Specificity	29.0 (36/124)	52.2 (36/69)	96.3 (77/80)
PPV	42.5 (65/153)	59.3 (48/81)	88.9 (24/27)
NPV	80.0 (36/45)	80.0 (36/45)	92.8 (77/83)
Accuracy	51.0 (101/198)	66.7 (84/126)	91.8 (101/110)

FNA: fine needle aspiration; FS: frozen section; PPV: positive predictive value; NPV: negative predictive value.

Note: raw data are in parenthesis.

∗Inadequate cytology results excluded, indeterminate, suspicious for malignancy and malignant cytology results considered positive.

^†^Inadequate and indeterminate cytology results excluded.

^‡^46 nodules excluded due to deferred results on FS.
